# “That’s Definitely a Red Flag”: Sexual Violence Risk Perception by Men who have Sex with Men Using Dating and Sexual Networking Apps

**DOI:** 10.1007/s12119-024-10301-4

**Published:** 2024-12-13

**Authors:** Megan Korovich, Alexandra Nicoletti, Marta Bettinelli, Faith Shank, D. J. Angelone, Meredith C. Jones

**Affiliations:** Department of Psychology, Rowan University, 201 Mullica Hill Road, Glassboro, NJ 08028 USA

**Keywords:** Men who have sex with men, MSM, Gay men, Bisexual men, Dating apps, Sexual violence

## Abstract

Sexual violence (SV) victimization is a growing concern for men who have sex with men (MSM), given their high risk for SV relative to heterosexual peers. MSM frequently utilize dating and sexual networking (DSN) apps to meet potential partners, which also puts them at a higher risk of victimization. Understanding the connection between DSN apps and SV victimization for MSM can inform the development of prevention interventions. The aim of this study was to examine partner characteristics MSM perceive as associated with SV risk on DSN apps. We conducted two virtual semi-structured focus groups with 14 MSM between the ages of 18 and 30. Group facilitators queried participants about their experiences with DSN apps and attributes of potentially risky partners. Three levels of perceived SV risk cues emerged as primary themes: yellow flags (attributes used to determine continued engagement), orange flags (caution or warning signs), and red flags (clear indications of danger). MSM described how they use these “flags” to evaluate SV risk when considering whether to interact with potential partners on DSN apps. Future research should assess whether these attributes are indeed associated with SV perpetration and whether strengthening skills for safely navigating DSN app interactions can reduce victimization for MSM.

## Introduction

Sexual violence (SV) is a major public health issue in the United States that affects people of all genders, sexual orientations, and ages. SV refers to sexual acts that are committed or attempted by another person without freely given consent of the victim or against someone who is unable to consent or refuse (Basile et al., 2014). Such acts can include any type of oral, anal, or vaginal contact and/or penetration in which the perpetrator uses force, intimidation, coercion, and/or other means (e.g., purposeful intoxication) to impose sexual contact on their victim (Cantor et al., 2015). While much of the literature focuses on heterosexual individuals, especially women, SV may be more prevalent among sexual minorities. The existing body of research suggests that sexual minorities—more specifically, men who have sex with men (MSM)—are up to 5 times more likely to experience SV as compared to their heterosexual peers (Balsam et al., 2005; Choi et al., 2017).

SV is associated with chronic negative mental and physical health outcomes (Choudhary et al., 2012); however, research on the consequences of SV has focused on female victims, while understanding of the effects on male victims lags behind (Tewksbury, 2007). The existing literature suggests that adult men who report having been sexually assaulted are more likely to report poorer physical and mental health than men who do not report SV victimization. Moreover, frequent psychological concerns reported by men following SV victimization include depression, anxiety, self-medication attempts (e.g., abuse of alcohol or illicit drugs), and decreased levels of self-esteem (Tewksbury, 2007).

Over the past two decades, dating and sexual networking (DSN) apps have become one of the most popular venues for seeking intimate partners. Apps like Tinder and Grindr boast millions of users (Filice et al., 2022; Goedel & Duncan, 2015), including MSM. In fact, 63.6% of MSM report meeting intimate partners online or on via apps in the past year (Goedel & Duncan, 2015; Janulis et al., 2017). Online dating, however; can be a risk factor for SV. For example, the use of DSN apps is associated with increased instances of unprotected, and frequently unwanted, sexual encounters (Choi et al., 2017). In fact, 11% of men reported an unwanted sexual experience with someone met on a dating site or app (Powell & Henry, 2016). Evidence also suggests a recent dramatic increase in SV perpetrated by partners met online and in dating apps, where 15% of victims were men (National Crime Agency, 2016). Taken together, those who use DSN apps are at a higher risk of SV victimization than those who do not use these services (Choi et al., 2016; Shapiro et al., 2017). Since MSM frequently utilize these apps, it is important to examine how men are making sexual decisions from their interactions on dating apps.

Much of this risk for SVvictimization can be explained by the theory of planned behavior (TPB; Ajzen, 1991, 2012), which demonstrates how a perpetrator’s behaviors are determined by their process of evaluating risks and benefits of situations that could influence their desired outcomes. Thus, SV may be prevalent in users who connect on DSN apps for a variety of reasons. First, dating apps in general create norms with expectations of sexual behaviors, whether that be sexual messaging or eventual meet ups (Miller, 2015). These apps were created to increase accessibility and ease of meeting potential partners, and most individuals using DSN apps would think and feel similarly. A potential perpetrator might not be flagged immediately for inappropriate sexual forwardness in the same way they would if meeting someone in-person.

Next, DSN apps provide a sense of anonymity for users, which may contribute to perceived power over sharing personal and private information on online spaces (Choi et al., 2017). Many app users may misrepresent their identity or forgo sharing personal details, post a photo without a face, or only share their location (Bauermeister et al., 2010). DSN apps also create access to potential perpetrators, particularly since app users may have heightened expectations for sex and meet ups in private locations (Choi et al., 2017). Finally, MSM are often under the influence of alcohol or other drugs both when using dating apps as well as when they meet with other users offline (Choi et al., 2016). This may increase a perpetrator’s perceived behavioral control over a potential victim, increasing their motivation to commit harm.

DSN apps present a unique opportunity for MSM to detect SV risk cues (Miller, 2015). In fact, several researchers have suggested that MSM utilize information on profiles to make decisions about their safety and potential sexual behaviors with that partner (Albury & Byron, 2016; Padgett, 2007). For example, if an online user only displays photos with a torso, abs, and no face, this can be associated with sexual risk (Winetrobe et al., 2014). If a profile only displays one picture, or the user is not willing to send a face photo within the online conversation, this is associated with inauthenticity (Albury & Byron, 2016). Minimal, nonexistent, or grammatically incorrect bios are also associated with untrustworthiness (Jozsa et al., 2021). However, at present, there is minimal research that has explored other forms of “red flags” on DSN apps. Given the prevalence of SV on DSN apps, it is important to understand users’ ability to detect risky cues and highlight the connection between stimuli on other users’ profiles to develop tools for prevention.

### Present Study

Understanding the connection between DSN apps and SV victimization, especially for vulnerable populations like MSM, can inform the development of prevention interventions. At present, there is a lack of information about how MSM use DSN apps to detect SV risk. However, given the popularity and growth of DSN app use among MSM, it is important to understand risk cues and “red flags” in these online spaces. Evaluating perceived SV risk cues can help inform SV prevention for online spaces, as well as educate MSM about navigating communication to detect potential risk on DSN apps. As such, we examined the experiences of MSM on DSN apps, including characteristics of potential partners and profile stimuli that are associated with perceptions of risk, or “red flags.”

## Materials and Methods

The data presented in this study comes from a larger project examining SV risk perception among MSM. The purpose of the focus groups was to gather information about MSM’s experiences with DSN apps, and to identify participants’ perceptions of both positive and negative attributes of potential partners to be used in the development of a novel laboratory analogue of risk perception for MSM (Angelone et al., 2024). We are developing this paradigm to be used in SV prevention endeavors, especially the study of risk detection under the effects of alcohol intoxication, and as a tool to educate participants about navigating SV risk and communication in DSN-app environments (e.g., participants could be reinforced for “good” decisions, and educated about risky decisions, in real time).

### Procedure of the Study

All study procedures received ethical approval from the institutional review board of the corresponding author’s university. Participants were recruited through advertisements posted on social media platforms (i.e., Twitter, Craigslist), as well as in local businesses in the greater Philadelphia region, a large metropolitan area in the United States. All participants completed an online screening to determine eligibility and availability for participation. Before joining a focus group, participants met individually with researchers through Zoom to go through the informed consent, as well as audio recording consent forms, which were then electronically signed through Qualtrics.

Data was collected through online focus groups which were conducted virtually in Spring 2022, three weeks apart, and were co-led by two clinical psychology doctoral students. Each session lasted approximately two hours, and participants were compensated with $40 gift cards. Only audio data was recorded from the focus groups to which transcriptions were made by the research team. Participants were discouraged from disclosing identifying information during the focus groups, but any identifiers were stricken from the transcripts and participants were given generic ID labels. Once anonymized, the transcripts were uploaded into Dedoose software (Dedoose, 2021) for coding and analyses.

### Participants

Eligibility for this study included: 1) between the ages of 18–30, 2) identification as a man, 3) sexual contact with a man in the past 12 months, and 4) have used a dating or sexual networking app in the past 12 months. We conducted two focus groups with 14 participants who met the inclusion criteria. The average age of participants was 23.5 years (SD = 1.5). Participants self-reported their sexual orientation as bisexual (42.9%, n = 6), gay (21.4%, n = 3), or not reported (35.7%, n = 5). Out of the participants who disclosed their race and ethnicity, 7 were Black (50.0%), and 2 were white (14.3%).

### Measures

Focus groups were conducted in a semi-structured format, and researchers were provided guided prompts to utilize within sessions. The guided prompts were developed by the research team, reviewed by project consultants, and revised according to team feedback. Items were determined as necessary for inclusion based on needs of laboratory analogue development and based on the current literature about SV risk in dating apps. A variety of questions (Table 1) were used to guide the focus groups to address what participants thought about dating apps, how they evaluated potential partners, and any identifiable patterns of victimization risk.

## Results

We used thematic analysis on the transcription data collected during the focus groups. Thematic analysis allowed for participant data to guide the themes that emerged, not the questions that were created by the research team. Major themes that arose during the analysis were categorized into perceptions of severity of risk. These were described as either 1) “yellow flags”, or factors that could decrease desirability but not necessarily cause concern of safety; 2) more ambiguous “orange flags” that suggest caution may be warranted for potentially unsafe behaviors; and 3) “red flags”, or clear indications of “dangerousness” or risk for victimization that constitute an “immediate dealbreaker” (i.e., reason to disengage) from a potential partner.

### General Feedback

We found that MSM reported a large number of negative experiences on dating apps. Many participants considered the apps to be “bad”, “terrible”, and “like complete shit.” For example, some apps such as Grindr reportedly foster “toxic” cultures making it difficult for users to form connections with others. Participants endorsed that dating apps have few opportunities to find long-term or monogamous partners but some platforms (i.e., OKCupid) provide more opportunity for dating compared to other apps. Many believed that the only intention of being on dating apps is to find someone to engage in hook-ups, or non-serious sexual commitments, and that most potential partners are not interested in their personal needs. Some participants discussed more positive experiences with DSN apps and stated that they have “personally worked well.” They found that the platforms were efficient and “a great way to get to know others in an ‘interview’ type fashion, where you can make sure a partner is meeting all of one’s personal requirements before meeting them”. Another participant believed that DSN apps provide better privacy and security as opposed to meeting potential partners in public, which conflicted with others’ views of finding it difficult to trust others on dating apps.

### Yellow Flags

Not unexpectedly, many participants discussed the importance of appearance for determining the attractiveness of potential partners. However, participants noted that to receive higher engagement from potential partners, they believed they should subscribe to specific beauty ideals:

I feel like a lot of stuff still adheres to a lot of cis/hetero norms in terms of desirability… and like standard or typical beauty standard stuff for men and all of that kind of stuff for body. (20-year-old, Transgender, Gay MSM)

One participant described discontent that apps such as Grindr have “tribe sections” where individuals can join based on their preferences for appearances, stating that “It’s bad, but like the body type is a big thing, like on Grindr, they have the tribe section and it’s unhealthy, but it is what a lot of people look at” (25-year-old MSM).

Other subthemes identified by participants that may affect desirability or attractiveness include age, employment, and disclosed HIV status. These factors appear to vary more on an individual basis. One participant suggested that desirability could be influenced by a potential partner’s job or their financial status, depending on the situation:

I think that a lot of the time, again, a job doesn’t really matter because a lot of it is just hookup culture. And so, like income level one that I don’t think people really look at that unless they are actually trying to, like, be in a relationship. (20-year-old, Transgender, Gay MSM)

Participants mentioned personal preferences of wanting someone more mature, and better able to focus on their needs in a relationship, as “age is also a very big determinant. You don’t want young blood, which is more energetic and outgoing. You just want someone who’s kind of personalized and focused towards ensuring that they’re bettering you” (23-year-old, White, Gay MSM).

Apps such as Grindr provide options for individuals to disclose their HIV status on their profile, and even mark themselves as “undetectable.” This function can be used to consider potential partners, but it was suggested it could also lead someone to be perceived as “less desirable” because individuals have the potential to lie about their HIV status or detectability.

I think that’s very important because like on Grindr, it has the HIV thing and you can say, but I feel like a lot of people might lie about that if they do have it, because it’s not like something you’d be honest about I feel like especially, if you’re on Grindr, which is an app for kind of shady practices to begin with. (25-year-old MSM)

### Orange Flags

Participants reported many aspects of potential partners that would raise more significant concerns in terms of deciding whether or not to pursue a hook-up. Facial expressions used in profile pictures were described as one way to detect personality traits, or whether someone was “toxic” or “threatening”:

A person with a more serious look in their profile picture looks to me, really speaks or really expresses some toxic traits indirectly, unlike a person who is lively and smiles in their profile. (22-year-old, Black, Bisexual MSM)

One negative profile feature that most participants noted included the use of “torso-only” photographs of potential partners. Not only did they indicate these kinds of profile pictures made it difficult to determine desirability, torso-only photographs were also cited as an indicator of a toxic personality for many participants. For example:

A lot of Grindr pictures are like just torso pictures, which is bad because I want to see the face. I want to see someone’s face, not their body kind of situation. Like if people just post their body. I’m like, I really don’t know if I’m attracted to you. I can’t really tell by your body. (25-year-old MSM)

Some participants discussed how the culture of apps such as Grindr facilitates narcissism because of the emphasis on profile views and message counts, which they did not see as a positive trait in potential partners. Participants noted that they viewed individuals as narcissistic if their profile descriptions came across as self-centered, and if they mentioned themselves frequently throughout conversations:

They’ll ask you a question and they don’t really care or respond to your response. They ask you it so that they can start talking about that topic for themselves. And that like that is one big thing. I’m like, oh, OK, you really don’t care about anything I have to say. (20-year-old, Transgender, Gay MSM)

When discussing profile characteristics, they believed were indicative of “toxic personalities”, the issue of a lack of empathy was mentioned by multiple participants. They reported believing there is a culture of little to no empathy among DSN app users. In fact, some participants reported that users may have fewer matches if they are outwardly empathetic, and believe many potential partners are looking for someone who will be “mean” to them:

Empathy is kind of a void in Grindr, but at the same time, it’s more like they don’t want emotional (inaudible). The risk factor is you won’t get as much matches if you’re empathetic, but at the same time, if you want relationships, you kind of have to be empathetic. It’s more risky not being empathetic like that stance, I guess. (25-year-old MSM)

One participant discussed their personal concerns with potential partners who detail in their descriptions that they are not interested in bisexual men. This brings up concerns about the partner endorsing internalized homonegativity, and not being accepting of non-cisgender men as well:

With like gay dudes who are like no bi dudes it’s like, it just is like you, to me, it kind of conveys is like, oh, you haven’t decided yet or you don’t really like guys. You mostly like girls or you’re not really part of the like MSM community because you like other genders besides dudes. It’s just, yeah, it’s very exclusive. And then again, for me, because I’m also not like a cis gay guy, it is meaning like, oh, they don’t like bi dudes, they probably don’t like trans people. (20-year-old, Transgender, Gay MSM)

### Red Flags

Participants also listed factors that were “immediate deal-breakers” for them about potential partners. One participant cited firsthand experiences of difficulty with potential partners due to their engagement in sex work. They reported often getting rejected because individuals were not comfortable with someone having multiple partners, or they might get propositioned for “free services,” which they find unacceptable. Another participant disclosed never becoming involved with anyone who identifies as a “sugar daddy.” They stated that this raises red flags as these individuals are often lying and using the title to exploit users on the app.

People that put sugar daddy in their bios. Never, I’d never trust that. Because it’s always fake. It’s rarely ever real and most of the time it’s just like they say that in order to get sexual like stuff out of you, whether it be like nudes or hookups or whatever. (20-year-old, Transgender, Gay MSM)

Additionally, most participants agreed that having a previous rape or sexual assault charge or accusation would constitute an immediate deal breaker. However, it was discussed that this information is typically only discovered through word of mouth from friends and acquaintances.

Visible patriotism or symbols of American cultural hypermasculinity in profiles was another indicator of dangerousness to participants. Potential partners who had images such as an American flag or a pickup truck in their profile pictures were not viewed positively. Participants reported associating patriotism and hypermasculinity with aggression toward the LGBTIA + community and potential internalized homonegativity. They also believe these individuals would not be friendly or welcoming to individuals that do not fit in with the normative gay community (e.g., bisexual and trans men).

If they have, like a lot of U.S flags or, you know, posed with U.S. flags or whatever, like that is also a red flag to me... just because like a lot of aggressive patriotism in this country pertaining to that, it also comes with aggressive isms and phobias and stuff so like people being super, super into their U.S. flag and super, super into the U.S. are usually, in my experience, either racist or sexist or misogynistic transphobic queer phobic or just of all of those. (20-year-old, Transgender, Gay MSM)

Participants also discussed the frequency of unsolicited genital pictures across many of the DSN apps that contributed to perceiving potential partners as dangerous. They noted on certain apps, individuals can begin conversation with any potential partner with an uncensored picture, while other apps restrict this behavior. Participants cited this as a major concern and problematic issue with DSN apps because they are being forced to view unsolicited photos. One participant reported that they believed explicit pictures shared without consent can be construed as a form of violence.

When I asked for a face pic, they’re like dick the first please. They ask for like my dick pic first before they’ll send me a face pic and that’s kind of problematic. I don’t know if I like you. But I’m going to get your private photos. (25-year-old MSM)

Participants also stated that while they are open to different kinks, some individuals will discuss their kinks in a forceful manner that makes them feel as though they would be forced to engage in such behaviors if they were to meet in person.

If I get a weird message where it’s like a weird proposition, like a lot of the messages you get on Grindr that are like they’re kinks, but the way they’re portrayed sounds like they’d still do them if you weren’t into it if you met them. (25-year-old MSM)

Relatedly, participants viewed potential partners as dangerous when their conversations showed indications of intimidation and control. Many reported how a potential partner talks about their ex-partners to be revealing of this as well. Discussing ex-partners in a demeaning or dehumanizing manner made participants feel a potential partner would be unsafe.

Like just talking about like exes still wanting to be with them or being jealous of like them or whatever that kind of stuff like in that way is like, OK, they might like it doesn’t it doesn’t say that they’re going to manipulate me, but clearly, they think that like they are hot shit and like they are THE hot shit in whatever relationship we’re going to have. (20-year-old, Transgender, Gay MSM)

### Participant Suggestions

At the end of each focus group, participants were asked to offer suggestions to make DSN apps safer for users. Overall, participants believed that more research should be conducted to bring awareness to SV occurring among DSN app users. Further, two suggestions related to increasing security in individual profiles. Participants believed there should be required verifications when creating profiles regarding sexual assault charges and HIV status, to remove the ability for individuals to lie in their profiles and personal interactions. These verifications would be intended to protect app users so that they can be aware of their risk level when engaging with potential partners.

## Discussion

The purpose of this study was to examine how MSM engage DSN apps and to highlight their perceptions of both the positive and negative qualities of potential partners, including characteristics which might suggest SV risk. Our findings highlight that MSM heavily utilize appearance and profile pictures in their evaluations of potential partners, not only for attraction, but for assessing SV risk. While there is limited information provided in a DSN app profile, there are several key themes that influence the determination of potential risk. Yellow flags identified factors that may be off-putting but did not bring up concerns of safety with a potential partner. Orange flags indicated individual factors that had the potential for safety issues but could be mediated by other elements. Red flags were definitive aspects of a partner that made them seem dangerous or unsafe to pursue a relationship with. Overall, these findings are consistent with prior evidence that suggests MSM use information from dating profiles to make decisions about their safety (Albury & Byron, 2016; Padgett, 2007).

Unsurprisingly, a focus on attractiveness of the individual and other characteristics of desirability play a dominant role. These “yellow flag” attributes, such as physical appearance, maturity, and financial status play a key role in the decision to continue engaging with other app users and potentially meeting offline. Participants acknowledged that some individuals may “lie” or otherwise exaggerate their attributes, but these elements were of little to no concern for their safety. That said, it is unclear whether these attributes ultimately translate into actual safety risks in real-world meetings; however, MSM tend to view these yellow flags as wholly unrelated to risk for SV.

MSM reported utilizing a variety of profile elements to detect possible risk for SV. These “orange flags” include facial expressions that may suggest a lack of empathy. In addition, descriptions of sex work or sugar daddy status led many men to proceed cautiously. These anecdotal reports also seem to be associated with data suggesting that MSM are less trusting of older men and large age gaps (Jozsa et al., 2021). Finally, another orange flag was “torso-only” images. Participants reported that the lack of one’s face on a profile photo or the appearance of fake images may be cause for concern. Indeed, research suggests that torso-only photos can be a cue for sexual risk (Winetrobe et al., 2014). MSM report a lack of trust in profiles with only torsos (Jozsa et al., 2021) and greater trust in a potential partner if they included real photos (Albury & Byron, 2016).

Regarding clear indicators of risk, MSM highlighted a long list of profile elements that suggest a “red flag” approach to SV risk detection. One point of contention was profiles that include portrayals of patriotism which may suggest the potential for aggression towards LGBTIA + individuals. MSM also reported that unsolicited genital pics or forced kinks are considered a form of sexual harassment and such callousness overriding the informal rules about “not including faces” or “only sending such images after establishing intimacy” are deal breakers (Albury & Byron, 2016). Finally, some MSM reported the demonstration of behaviors suggesting intimidation or control serve as immediate signs of dangerousness. Indeed, research suggests that there are a variety of behavioral qualities that MSM use to determine untrustworthiness and potential partners who demonstrate a demanding or hostile personality are deemed untrustworthy (Jozsa et al., 2021). Interestingly, while research suggests substance use is closely associated with SV (Abbey et al., 2001), participants did not highlight this as a concern. Rather, this seems to be an acceptable part of hookup culture among MSM.

While these data provide a glimpse into the sexual decision making of MSM app users, our study is not without some limitations. First, all participants were recruited from the larger Philadelphia metropolitan area. The dating and sexual networking of individuals from this region may differ quite dramatically from individuals located elsewhere. As such, future researchers would be wise to involve men from other parts of the country, including both rural and urban locales. Additionally, most of our participants reported a racial/ethnic minority status and relatively younger age. While this can be considered a strength, our study cannot serve as a snapshot of all MSM. For example, much research has demonstrated the fetishism of certain racial/ethnic individuals, especially Black and younger men. Thus, these individuals may have different perspectives on signs of desirability and SV risk among other DSN users. Finally, the data for this study was collected between Fall 2021 and Spring 2022, overlapping considerably with the COVID-19 pandemic. One effect of the pandemic led to increased use of technology or online resources within individual’s sexual activities (Lehmiller et al., 2020), which could include novice users who may be less well versed in identifying risk in DSN apps. Thus, it is unclear whether these themes would be replicated in future dating scenarios given the well-documented changes to interpersonal relationships both during and because of the pandemic.

In the end, we hope this initial exploratory work can guide future researchers to develop more comprehensive studies of DSN app users and their sexual decision making. We believe these themes can be further explored in a large-scale nationwide survey to identify the types of risk cues men perceive and the extent to which different types of men do so. For example, are younger men more likely to dismiss certain cues relative to older men? Are there racial/ethnic differences in the way men engage potential partners in these apps? Future research should explore whether risk perception cues described by MSM in this study are associated with actual SV perpetration, which can inform prevention interventions aimed at teaching MSM to recognize such risk cues and develop skills to safely navigate DSN app use. Finally, we also believe clinicians can leverage this understanding to better educate MSM about risk perception and safe dating practices, particularly around red flags such as aggressive behaviors or troubling profile content. Additionally, this study suggests the need for tailored interventions that consider the diverse experiences of MSM across different demographics and regions. By fostering awareness and communication about these risk cues, clinicians can enhance the safety and well-being of their clients in the context of online dating.

## Data Availability

The data that support the findings of this study are available from the corresponding author upon reasonable request.

## Appendix

See Table 1.

**Table 1:** Focus Group Guide

Question	Follow-Up Prompt
In general, what do you think about dating and sexual networking apps?	*Apps like Grindr, Tinder, things like that*
What do you think most 18–29 year old gay and bisexual men would find desirable/attractive in regard to online dating profiles?	*For example, jobs, age, body features, relationship status, HIV status, substance use…*
What would most 18–29-year-old gay and bisexual men find as dealbreakers on a profile?	
We’re studying how men experience and navigate sexual violence. Is that something you ever think about or deal with as you use dating apps?	
In general, when you’re getting to know a potential new partner, how do you know when they’re potentially dangerous?	*What elements might appear on his profile that would demonstrate risk?* *What does he do or say that would cue you in?*
The research says certain characteristics might be associated with ***sexual violence risk*** such as: Internalized homonegativity Toxic personality traits Difficulty with alcohol or drug use Potential to take advantage Likes to intimidate and control Previous perpetration of sexual assault	*How would you know that a guy was demonstrating these risk factors in a profile or conversation?*

## References

[CR1] Abbey, A., Zawacki, T., Buck, P. O., Clinton, A. M., & McAusian, P. (2001). Alcohol and sexual assault. *Alcohol Research and Health,**25*(1), 43–51. https://pmc.ncbi.nlm.nih.gov/articles/PMC4484576/11496965 PMC4484576

[CR2] Albury, K., & Byron, P. (2016). Safe on my phone? Same-sex attracted young people’s negotiations of intimacy, visibility, and risk on digital hook-up apps. *Social Media + Society*. 10.1177/2056305116672887

[CR21] Angelone, D. J., Mitchell, D., Wells, B., Korovich, M., Nicoletti, A., & Fife, D. (2024). Assessment of sexual violence risk perception in men who have sex with men: Proposal for the development and validation of “G-Date”. *JMIR Research Protocols*, *13*, e57600. 10.2196/57600 39159453 10.2196/57600 PMC11369526

[CR3] Balsam, K. F., Rothblum, E. D., & Beauchaine, T. P. (2005). Victimization over the life span: A comparison of lesbian, gay, bisexual, and heterosexual siblings. *Journal of Consulting and Clinical Psychology,**73*(3), 477–487. 10.1037/0022-006X.73.3.477 15982145 10.1037/0022-006X.73.3.477

[CR4] Basile, K. C., Smith, S. G., Breiding, M. J., Black, M. C., & Mahendra, R. R. (2014). *Sexual violence surveillance: Uniform definitions and recommended data elements, Version 2.0.* Atlanta, GA: National Center for Injury Prevention and Control, Centers for Disease Control and Prevention. https://www.cdc.gov/violenceprevention/pdf/sv_surveillance_definitionsl-2009-a.pdf

[CR5] Bauermeister, J. A., Giguere, R., Carballo-Diéguez, A., Ventuneac, A., & Eisenberg, A. (2010). Perceived risks and protective strategies employed by young men who have sex with men (YMSM) when seeking online sexual partners. *Journal of Health Communication,**15*(6), 679–690. 10.1080/10810730.2010.499597 20812127 10.1080/10810730.2010.499597 PMC2933919

[CR6] Cantor, D., Fisher, B., Chibnall, S., Townsend, R., Lee, H., Bruce, C., & Thomas, G. (2015). Report on the AAU campus climate survey on sexual assault and sexual misconduct. *Association of American Universities*, *21*. https://assets.documentcloud.org/documents/2674806/University-of-Virginia-Climate-Survey.pdf

[CR7] Choi, E. P. H., Wong, J. Y. H., & Fong, D. Y. T. (2017). The use of social networking applications of smartphone and associated sexual risks in lesbian, gay, bisexual, and transgender populations: A systematic review. *AIDS Care,**29*(2), 145–155. 10.1080/09540121.2016.1211606 27454158 10.1080/09540121.2016.1211606

[CR8] Choudhary, E., Smith, M., & Bossarte, R. M. (2012). Depression, anxiety, and symptom profiles among female and male victims of sexual violence. *American Journal of Men’s Health,**6*(1), 28–36. 10.1177/1557988311414045 10.1177/1557988311414045 22105064

[CR9] Goedel, W. C., & Duncan, D. T. (2015). Geosocial-networking app usage patterns of gay, bisexual, and other men who have sex with men: Survey among users of Grindr, a mobile dating app. *JMIR Public Health and Surveillance*. 10.2196/publichealth.4353 10.2196/publichealth.4353 PMC4869243 27227127

[CR10] Janulis, P., Feinstein, B. A., Phillips, G., Newcomb, M. E., Birkett, M., & Mustanski, B. (2017). Sexual partner typologies and the association between drug use and sexual risk behavior among young men who have sex with men. *Archives of Sexual Behavior*. 10.1007/s10508-016-0909-x 10.1007/s10508-016-0909-x PMC5554732 28194606

[CR11] Jozsa, K., Kraus, A., Korpak, A. K., Birnholtz, J., Moskowitz, D. A., & Macapagal, K. (2021). “Safe behind my screen”: Adolescent sexual minority males’ perceptions of safety and trustworthiness on geosocial and social networking apps. *Archives of Sexual Behavior,**50*(7), 2965–2980. 10.1007/s10508-021-01962-5 34581948 10.1007/s10508-021-01962-5

[CR12] Lehmiller, J. J., Garcia, J. R., Gesselman, A. M., & Mark, K. P. (2020). Less sex, but more sexual diversity: Changes in sexual behavior during the COVID-19 coronavirus pandemic. *Leisure Sciences,**43*(1–2), 295–304. 10.1080/01490400.2020.1774016

[CR13] Miller, B. (2015). “They’re the modern-day gay bar”: Exploring the uses and gratifications of social networks for men who have sex with men. *Computers in Human Behavior,**51*, 476–482. 10.1016/j.chb.2015.05.023

[CR14] National Crime Agency. (2016, February 7). *Emerging new threat in online dating: Initial trends in internet dating-initiated serious sexual assaults*.http://www.nationalcrimeagency.gov.uk/

[CR15] Padgett, P. M. (2007). Personal safety and sexual safety for women using online personal ads. *Sexuality Research & Social Policy,**4*(2), 27–37. 10.1525/srsp.2007.4.2.27

[CR16] Powell, A., & Henry, N. (2016). Policing technology-facilitated sexual violence against adult victims: Police and service sector perspectives. *Policing and Society*. 10.1080/10439463.2016.1154964

[CR17] Shapiro, G. K., Tatar, O., Sutton, A., Fisher, W., Naz, A., Perez, S., & Rosberger, Z. (2017). Correlates of Tinder use and risky sexual behaviors in young adults. *Cyberpsychology, Behavior, and Social Networking,**20*(12), 727–734. 10.1089/cyber.2017.0279 29211500 10.1089/cyber.2017.0279

[CR18] Tewksbury, R. (2007). Effects of sexual assaults on men: Physical, mental and sexual consequences. *International Journal of Men’s Health,**6*(1), 22–35. 10.3149/jmh.0601.22

[CR19] Dedoose Version 9.0.17, web application for managing, analyzing, and presenting qualitative and mixed method research data. (2021). Los Angeles, CA, USA: SocioCulturalResearch Consultants LLC. Retrieved from www.dedoose.com.

[CR20] Winetrobe, H., Rice, E., Bauermeister, J., Petering, R., & Holloway, I. W. (2014). Associations of unprotected anal intercourse with Grindr-met partners among Grindr-using young men who have sex with men in Los Angeles. *AIDS Care,**26*(10), 1303–1308. 10.1080/09540121.2014.911811 24754563 10.1080/09540121.2014.911811

